# The contribution of executive functions and emotion comprehension skills to the development of pragmatic competence in 5–8-year-old children

**DOI:** 10.3389/fpsyg.2025.1659576

**Published:** 2025-09-25

**Authors:** Ekaterina Oshchepkova, Arina Shatskaya, Kristina Tarasova

**Affiliations:** Faculty of Psychology, Lomonosov Moscow State University, Moscow, Russia

**Keywords:** pragmatics, executive functions, test of emotion comprehension, pragmatic development, preschool age, typically developing children, regression models

## Abstract

A child’s pragmatic competence reflects both their social and communicative abilities, as well as their understanding of indirect meaning in words, utterances or discourse. This has led to a growing interest in the development of pragmatics in children. While the contribution of cognitive and emotional developmental aspects to pragmatic competence in general has been explored, the role of the emotion comprehension (EC) and executive functions (EF) in different pragmatic skills is still insufficiently studied. The purpose of this study was to investigate the impact of children’s EF, and EC skills, on four core aspects of pragmatic competence (understanding, production, nonverbal means and communication). Participants were children (*N* = 1,842) aged 59–96 months (*M* = 73.51, SD = 9.0) and their teachers. Children completed tests assessing their EF (NEPSY-II) and understanding of emotions (Test of Emotion Comprehension), and their teachers completed a questionnaire of the children’s pragmatic competence. Through comparison of baseline and extended regression models, it was shown that although EF contribute significantly to all aspects of pragmatic competence, the connections with EC remain at a correlational level. Their contribution to pragmatic competence is not confirmed. These findings can support the development of programs to enhance children’s pragmatic competence, targeting educators, parents, and the children themselves.

## Introduction

Pragmatics, or language in social context, is a construct that has been studied for more than half a century and is analyzed, among other things, from the perspective of ontogenetic development (Pearson and de Villiers, 2006). The development of pragmatic competence (the ability to use language appropriately in a social context (Taguchi, 2009)) in ontogeny is an essential component of a child’s overall social-communicative development. When examining the relationships between a child’s pragmatic skills and other aspects of their cognitive and emotional development, it becomes apparent that pragmatic skills are heterogeneous and their interrelations vary depending on the aspect considered. In this study, according to the instrument used (PCQCh), the child’s pragmatic skills include: (1) the ability to understand language in context; (2) communication skills; (3) the use of nonverbal means of communication; (4) narrative skills in different genres.

### The relationship between children’s pragmatic competence and the executive functions

By executive functions (EF) we follow the concept of Friedman and Miyake (2017), understanding them as high-level cognitive processes that control lower-level processes. According to this model, EF include cognitive flexibility, inhibitory control, and working memory. Research shows that EF regulate a wide range of mental functions in children and make a significant contribution to their development in preschool age. The relationship between pragmatic competence and the development of EF has primarily been studied in samples of children with autism spectrum disorders (ASD) (Filipe et al., 2020). These studies found that well-developed EF mitigate the negative impact of ASD on the pragmatic aspect of language in children from 8 to 18 years (Cardillo et al., 2021). In particular, a strong positive influence of well-developed working memory and attention on pragmatic skills in children aged 2–8 years with autism has been noted (Howard et al., 2023). Studies on this relationship in normatively developing populations remain relatively rare, and the available findings are often inconsistent (Blain-Brière et al., 2014). Nevertheless, several studies have emphasized the leading contribution of working memory to higher levels of pragmatic competence in adults and children aged 5–9 years (Bambini et al., 2021; Filippova and Astington, 2008). Also noted is the contribution of inhibitory control (Chen et al., 2025) to the comprehension of non-literal language and irony, as well as the role of cognitive flexibility (Hung and Loh, 2020) in meaning interpretation, which also pertains to pragmatic competence. Thus, despite numerous studies and the general conclusion about the contribution of EF to socio-communicative skills, their contribution to the development of pragmatics and its individual aspects in preschoolers remains insufficiently studied.

### The relationship between children’s pragmatic competence and emotion comprehension

By emotion comprehension (EC) we follow the concept of Pons and Harris (2005). EC is the ability to understand “the nature, causes, and consequences of the emotional experience in the self and others” (Pons and Harris, 2019, p. 431). Children’s EC development passes through several stages or levels (Pons and Harris, 2005; Pons et al., 2004). These levels are: (1) the first “External” level at 3–5 years old. This level is associated with the formation of ideas about the external causes of emotions, that is, recognition of others’ emotions from facial expressions and understanding the influence of external circumstances on emotions. (2) The second is the “Mental” level between the ages of 4–5 and 6–7. Children begin to understand that personal beliefs, desires, and memories can trigger different emotions and that certain emotions can be hidden. (3) The third or” Reflective” level occurs at 6–7 to 9–10 years. Children learn to regulate their emotions, begin to better understand ambivalent (mixed) and hidden emotions, and discover the influence of moral norms on emotions (Pons and Harris, 2005; Pons et al., 2003). Pragmatic competence includes communicative skills, that is, the ability and willingness to engage in communication and interaction with others. It would be logical to assume that understanding the interlocutor’s emotions makes a significant contribution to the pragmatic competence. However, there are relatively few studies directly investigating the relationship between EC and pragmatic skills. In particular, Veraksa et al. (2019) provides clear evidence of a positive relationship between pragmatic narrative ability and EC in typical preschoolers. Curenton’s (2015) work shows how an emotion-focused task can reveal pragmatic competence. Pronina et al. (2021) suggest that these skills can be dissociated in intervention. In sum, pragmatic skill appears intertwined with EC during preschool (Spackman et al., 2006): children who are better at social language tend to understand emotions better, whereas those with pragmatic impairments (from ASD, ADHD, or DLD) often show social–emotional weaknesses as well (Carruthers et al., 2022; Wong et al., 2022). Several studies have shown that pragmatic skills contribute to EC, but not vice versa (Farina et al., 2007; Giménez-Dasí et al., 2013). It has also been shown that work with 3- to 4-year-old children focused on expanding their vocabulary with terms related to mental states and emotion labels significantly improved their emotion understanding, as well as their false-belief understanding (Ornaghi et al., 2011). As for precise studies of contribution of EC in children in their pragmatic skills, they are missing.

### Research relevance and aim

Thus, the impact of EF and EC on pragmatic skills in children remains understudied. This research question is very important not only for designing intervention programs for children with ASD and communication disorders but also for enhancing the social-communicative and pragmatic skills of typically developing children. The aim of the present study is to assess the contribution of EF and EC to key aspects of pragmatic competence in children aged 5–8 years.

## Methods and sample

### Sample

The sample included typically developing children aged 5–8 years from middle-class families, encompassing both monolingual Russian speakers and Russian-dominant bilinguals. Children who did not complete both testing sessions, as well as those whose parents did not provide written informed consent, were excluded from the study. Initially the sample included more than 2000 children from six regions of the Russian Federation. However, after excluding irrelevant teacher responses to the PCQCh, valid assessments remained for 1,842 children, including 910 boys and 932 girls. The children were aged between 59 and 96 months (*M* = 73.51, SD = 9.0).

### Instruments and procedure

To assess the pragmatic competence of children, the Pragmatic Competence Questionnaire for Children aged 5–8 years (PCQCh) (Oshchepkova et al., 2024) was used. This questionnaire was completed by the educators of the children attending their classes. It includes 20 items grouped into four scales: Scale 1. The ability to understand language in context (e.g., “The child understands from your intonation and facial expression that you are dissatisfied, demanding something, or, conversely, that you are pleased,” 6–30 points, Cronbach’s *α* = 0.811); Scale 2. Communication skills (e.g., “When communicating with relatives and familiar adults, the child shows interest and friendliness,” 5–25 points, Cronbach’s *α* = 0.795); Scale 3. Use of nonverbal means of communication (e.g., “The child uses gestures appropriate to the situation,” 3–15 points, Cronbach’s *α* = 0.845); Scale 4. Narrative skills in different genres (e.g., “The child invents his/her own coherent stories, with a beginning, a sequence of events, and an ending,” 3–15 points, Cronbach’s *α* = 0.856). Teachers evaluate how characteristic this behavior is for the child, from 1 (this behavior is not characteristic at all) to 5 (this behavior is always characteristic of the child).

To assess the level of EC, the TEC (Test of Emotion Comprehension) (Pons and Harris, 2004) was used. TEC is a tool that allows to capture child emotion understanding and measure nine components of EC: (1) emotion recognition, (2) external cause, (3) desire, (4) belief, (5) reminder, (6) regulation, (7) hidden, (8) mixed, and (9) morally based emotions. At the same time, it enables the distinction of three levels of emotion understanding: the external level, the mental level, and the reflective level. The test consists of 22 tasks. An accurate answer receives 1 point, and a wrong answer receives a zero point. The scores obtained for individual tasks are combined into four main indicators: three of them (External, Mental, and Reflective) correspond to the three levels of EC per the authors’ (Pons et al., 2004) theory, and the fourth indicator (Total score) is the sum of the points obtained from the first three indicators. The score can range from 0 to 3 for each of the three indicators. Accordingly, the total score for EC can range between 0 and 9. The total raw score is the sum of points for all 22 tasks, ranging from 0 to 22 points. The reliability of this instrument for Russian-speaking samples has been demonstrated previously (Bukhalenkova et al., 2024).

To assess EF, the NEPSY-II assessment battery was used (Korkman et al., 2007). Using this instrument, the following components were assessed in children: cognitive flexibility, inhibitory control, and working memory (both visual and verbal). To assess inhibitory control and cognitive flexibility, the Inhibition subtest (NEPSY-II) was used: in the task, the child first had to correctly name circles and squares, as well as upward and downward arrows, and then, following the rule, name them in reverse—circles as squares, squares as circles, and arrows in the opposite direction. Tasks performance correctness and time were assessed. Working memory was assessed through Sentence repetition (verbal working memory, maximum score—34) and Memory for designs (visual working memory, maximum score—120). The test had been translated into Russian, adapted for use with Russian-speaking children aged 5–8 years and showed high reliability (Veraksa et al., 2020).

### Procedure

The assessment was conducted individually with each child in a quiet and bright room of the kindergarten attended by the children. Two sessions were organized with each child, lasting 15–20 min. Children were free to stop the test at any time. All methods were presented to children in the same established order: at the first session, the TEC, and Memory for Designs subtest; at the second session, the Inhibition and the Sentence repetition subtests were carried out. Prior to the study, the testers completed specialized training sessions on the administration and scoring procedures of the specified methods. Subsequently, they were required to pass a certification exam, which involved submitting a video recording of themselves conducting and processing the tests. Their scores were compared against those assigned by experienced experts. Only testers who achieved an inter-rater reliability score of at least Cronbach’s *α* = 0.81 were certified to conduct further testing.

All the parents were informed about the study goals and gave written consent for their children’s participation in the research. The study was carried out in those educational institutions with which cooperation agreements were concluded, and parental consent was collected with the help of teachers working in the groups attended by the children.

### Statistical analysis

Preliminary analyses included simple Pearson and Spearman correlation analyses for main study variables. Additional Mann–Whitney test was then conducted to assess the significance of differences in pragmatic competence scales between boys and girls.

To identify the most significant predictors of pragmatic competence, we adopted a block-wise regression approach, ensuring that all necessary assumptions for linear regression were met. In our analysis, each of the four pragmatic competence scales was, in turn, used as the dependent variable. The predictors were organized into three distinct blocks: one for sex and age, one for EF components, and one for EC variables. In the first stage, we constructed a baseline Model 1 that included only the demographic predictors (sex and age). In the second stage, we developed two alternative extended models: in Model 2.1, the baseline model was supplemented with EF components, while in Model 2.2, predictors for EC were added. In the final stage, we built Model 3 by integrating both the EF and EC blocks along with the original demographic predictors.

After constructing all models for each of the four pragmatic competence scales, Model 2 (in two versions, Model 2.1 and Model 2.2) was compared with the baseline model using *R*^2^, and Model 3 was compared with Model 2. Based on these comparisons, the best-fitting model was selected. All analyses were conducted in Jamovi (version 2.3.16).

## Results

### Preliminary analyses

Supplementary Table 1 displays Pearson and Spearman correlations among study variables. The correlational analysis revealed significant (at the *p* < 0.05 level) associations between the pragmatic competence and age, sex (except for “Narrative skills”), Mental level, External level and Reflective levels of EC (except for “Use of nonverbal means of communication”), Cognitive flexibility, Inhibition (except for “Narrative skills”), Verbal working memory, and Visual working memory.

The Mann–Whitney test for identifying sex differences in the pragmatic competence scales revealed significant differences for three of the scales. Differences were significant in favor of girls for “Ability to understand language in context” (*U* = 379,769, *p* < 0.001, Rank biserial correlation = 0.1044), “Communication skills” (*U* = 379,024, *p* < 0.001, Rank biserial correlation = 0.1062), and “Use of nonverbal means” (*U* = 380,505, *p* < 0.001, Rank biserial correlation = 0.1027). For “Narrative skills,” no significant differences were found (*U* = 404,272, *p* = 0.081, Rank biserial correlation = 0.0467).

### Regression models for pragmatic competence scales

Preliminary checks of the predictors did not reveal any multicollinearity issues (VIF statistics ranged from 1.01 to 1.60). We conducted separate block-wise linear regression models for each of the four scales of pragmatic competence. The models provide standardized estimates.

#### Regression models for scale “the ability to understand language in context”

At the first stage, the baseline Model 1, which included sex and age as predictors, was found to be significant (adjusted *R*^2^ = 0.05, *F* = 40.6, df₁ = 2, df₂ = 1,567, *p* < 0.001). Both sex (*β* = 0.20, *t* = 4.18, *p* < 0.001) and age (*β* = 0.20, *t* = 8.19, *p* < 0.001) made a significant contribution.

At the second stage, the baseline model was first supplemented first with a block of EF components (Model 2.1). This resulted in a significant model (adjusted *R*^2^ = 0.118, *F* = 36.1, df₁ = 6, df₂ = 1,563, *p* < 0.001), with significant predictors being visual working memory (*β* = 0.13, *t* = 4.38, *p* < 0.001), verbal working memory (*β* = 0.20, *t* = 7.52, *p* < 0.001), and sex.

Next, Model 2.2 was constructed, which consisted of sex, age, and components of EC (adjusted *R*^2^ = 0.06, *F* = 21.4, df₁ = 5, df₂ = 1,564, *p* < 0.001). The significant predictors of this version of the model turned out to be sex, age, external level of EC (*β* = 0.08, *t* = 3.13, *p* = 0.002), and reflective level of EC (*β* = 0.07, *t* = 2.76, *p* = 0.006).

Finally, at the third stage, the model was expanded to include all three blocks: sex, age, EF components, and EC (Model 3). The resulting model was significant (adjusted *R*^2^ = 0.119, *F* = 24.5, df₁ = 9, df₂ = 1,560, *p* < 0.001). Significant predictors are visual working memory (*β* = 0.12, *t* = 3.91, *p* < 0.001), verbal working memory (*β* = 0.2, *t* = 7.26, *p* < 0.001), and sex.

A comparison between the baseline model and Model 2.1 showed a significant improvement in model quality (Δ*R*^2^ = 0.07, *F* = 32.24, *p* < 0.001). A comparison between the baseline model and Model 2.2 showed a significant improvement in model quality (Δ*R*^2^ = 0.01, *F* = 8.22, *p* < 0.001). A comparison between Model 2.1 and Model 3 showed no significant improvement (Δ*R*^2^ = 0.002, *F* = 1.21, *p* = 0.304). A comparison between Model 2.2 and Model 3 showed significant improvement (Δ*R*^2^ = 0.06, *F* = 26.59, *p* < 0.001).

Therefore, Model 3 was selected as the best-fitting model (see Table 1). Thus, the most significant predictors of the ability to understand language in context are verbal and visual working memory. However, without taking into account the EF components, the level of EC (external and reflective) also contribute to this aspect of pragmatic competence.

**Table 1:** Regression model of scale 1: the ability to understand language in context.

Predictor	Estimate	SE	*t*	*p*	Stand. estimate
Intercept	13.992	1.12178	12.473	<0.001	
Sex	0.861	0.22326	3.856	<0.001	0.1838
Age	0.0202	0.01559	1.296	0.195	0.0373
External level of EC	0.2062	0.18163	1.135	0.257	0.0291
Mental level of EC	0.1245	0.14425	0.863	0.388	0.0217
Reflective level of EC	0.1477	0.13715	1.077	0.282	0.0269
Visual working memory	0.0238	0.00608	3.914	<0.001	0.1173
Verbal working memory	0.1988	0.02736	7.266	<0.001	0.1985
Cognitive flexibility	0.0467	0.04134	1.13	0.259	0.0314
Inhibition	0.0503	0.03704	1.358	0.175	0.034

#### Regression models for scale “communication skills”

At the first stage, the baseline model, which included sex and age as predictors, was found to be significant (adjusted *R*^2^ = 0.03, *F* = 26.6, df₁ = 2, df₂ = 1,567, *p* < 0.001). Both sex (*β* = 0.186, *t* = 3.74, *p* < 0.001) and age (*β* = 0.161, *t* = 6.45, *p* < 0.001) made significant contributions.

At the second stage, the baseline model was first supplemented first with a block of EF components (Model 2.1), resulting in a significant model (adjusted *R*^2^ = 0.079, *F* = 23.5, df₁ = 6, df₂ = 1,563, *p* < 0.001). The significant predictors were visual working memory (*β* = 0.114, *t* = 3.81, *p* < 0.001), verbal working memory (*β* = 0.187, *t* = 6.76, *p* < 0.001), and sex.

Next, Model 2.2 was constructed, which consisted of sex, age, and components of EC (adjusted *R*^2^ = 0.04, *F* = 14, df₁ = 5, df₂ = 1,564, *p* < 0.001). The significant predictors of this version of the model turned out to be sex, age, and mental level of EC (*β* = 0.06, *t* = 2.62, *p* = 0.009).

Finally, at the third stage, the model was expanded to include all three blocks: sex, age, EF components, and EC (Model 3). The resulting model was significant (adjusted *R*^2^ = 0.080, *F* = 16.2, df₁ = 9, df₂ = 1,560, *p* < 0.001). Significant predictors in Model 3 included visual working memory (*β* = 0.102, *t* = 3.34, *p* < 0.001), verbal working memory (*β* = 0.185, *t* = 6.39, *p* < 0.001), the mental level of EC (*β* = 0.05, *t* = 1.98, *p* = 0.048), and sex.

A comparison of the baseline model and Model 2.1 revealed a significant improvement in model quality (Δ*R*^2^ = 0.049, *F* = 21.28, *p* < 0.001). A comparison of the baseline model and Model 2.2 revealed a significant improvement in model quality (Δ*R*^2^ = 0.01, *F* = 5.5, *p* < 0.001). The comparison between Model 2.1 and Model 3 showed no significant improvement (Δ*R*^2^ = 0.003, *F* = 1.54, *p* = 0.203). The comparison between Model 2.2 and Model 3 showed significant improvement (Δ*R*^2^ = 0.04, *F* = 18.1, *p* < 0.001).

Based on the largest *R*^2^, the best model turned out to be the Model 3 (Table 2). Thus, the most significant predictors of communication skills are visual and verbal working memory, and the mental level of EC.

**Table 2:** Regression model of scale 2: communication skills.

Predictor	Estimate	SE	*t*	*p*	Stand. estimate
Intercept	16.7283	1.1612	14.406	<0.001	
Sex	0.8256	0.23111	3.572	<0.001	0.1739
Age	0.0148	0.01614	0.916	0.36	0.027
External level of EC	0.0803	0.18802	0.427	0.669	0.0112
Mental level of EC	0.2957	0.14932	1.981	0.048	0.0508
Reflective level of EC	0.0611	0.14197	0.43	0.667	0.011
Visual working memory	0.021	0.0063	3.342	<0.001	0.1023
Verbal working memory	0.1881	0.02832	6.639	<0.001	0.1853
Cognitive flexibility	−0.0241	0.0428	−0.563	0.573	−0.016
Inhibition	0.0291	0.03834	0.76	0.447	0.0195

#### Regression models for scale “use of nonverbal means of communication”

At the first stage, the baseline model, which included sex and age as predictors, was significant (adjusted *R*^2^ = 0.0204, *F* = 17.32, df₁ = 2, df₂ = 1,567, *p* < 0.001). Both sex (*β* = 0.198, *t* = 3.95, *p* < 0.001) and age (*β* = 0.114, *t* = 4.57, *p* < 0.001) made significant contributions.

At the second stage, the baseline model was first supplemented first with a block of EF components (Model 2.1), resulting in a significant model (adjusted *R*^2^ = 0.047, *F* = 13.93, df₁ = 6, df₂ = 1,563, *p* < 0.001). Significant predictors included visual working memory (*β* = 0.09, *t* = 3.02, *p* = 0.003), verbal working memory (*β* = 0.132, *t* = 4.7, *p* < 0.001), and sex.

Next, Model 2.2 was constructed, which consisted of sex, age, and scores for EC (adjusted *R*^2^ = 0.0203, *F* = 7.5, df₁ = 5, df₂ = 1,564, *p* < 0.001). The significant predictors of this version of the model turned out to be sex and age.

Finally, at the third stage, the model was expanded to include all three blocks: sex, age, EF components, and level of EC (Model 3), yielding a significant model (adjusted *R*^2^ = 0.045, *F* = 9.32, df₁ = 9, df₂ = 1,560, *p* < 0.001). Significant predictors are visual working memory (*β* = 0.09, *t* = 2.97, *p* = 0.003), verbal working memory (*β* = 0.131, *t* = 4.72, *p* < 0.001), and sex.

A comparison of the baseline model and Model 2.1 showed a significant improvement in model quality (Δ*R*^2^ = 0.029, *F* = 11.98, *p* < 0.001). A comparison of the baseline model and Model 2.2 did not reveal a significant improvement in model quality (Δ*R*^2^ = 0.001, *F* = 0.95, *p* = 0.416). Comparing Model 2.1 and Model 3 did not reveal any significant improvement (Δ*R*^2^ = 0.000, *F* = 0.149, *p* = 0.930). Comparing Model 2.2 and Model 3 revealed significant improvement (Δ*R*^2^ = 0.03, *F* = 11.4, *p* < 0.001).

Based on the largest *R*^2^, Model 2.1 was selected as the best-fitting model (see Table 3). Thus, the most significant predictors of nonverbal means of communication are verbal and visual working memory.

**Table 3:** Regression model of scale 3: use of nonverbal means of communication.

Predictor	Estimate	SE	*t*	*p*	Stand. estimate
Intercept	8.87231	0.62896	14.106	<0.001	
Sex	0.47563	0.12596	3.776	<0.001	0.18677
Age	0.00633	0.00864	0.734	0.463	0.02151
Cognitive flexibility	−0.00793	0.02315	−0.343	0.732	−0.00981
Visual working memory	0.01017	0.00337	3.020	0.003	0.09213
Inhibition	0.02437	0.02093	1.165	0.244	0.03035
Verbal working memory	0.07238	0.01535	4.715	<0.001	0.13293

#### Regression models for scale “narrative skills in different genres”

At the first stage, the baseline model, which included sex and age as predictors, was significant (adjusted *R*^2^ = 0.032, *F* = 27.6, df₁ = 2, df₂ = 1,567, *p* < 0.001). Only age was a significant predictor (*β* = 0.18, *t* = 7.29, *p* < 0.001), while sex was not (*β* = 0.09, *t* = 1.82, *p* = 0.069).

At the second stage, the baseline model was first supplemented with a block of EF components (Model 2.1), resulting in a significant model (adjusted *R*^2^ = 0.064, *F* = 18.8, df₁ = 6, df₂ = 1,563, *p* < 0.001). Significant predictors included verbal working memory (*β* = 0.176, *t* = 6.29, *p* < 0.001) and age.

Next, Model 2.2 was constructed, which consisted of sex, age, and scores of EC (adjusted *R*^2^ = 0.034, *F* = 12.1, df₁ = 5, df₂ = 1,564, *p* < 0.001). The significant predictors of this version of the model turned out to be age.

Finally, at the third stage, the model was expanded to include all three blocks: sex, age, EF components, and EC scores (Model 3), yielding a significant model (adjusted *R*^2^ = 0.063, *F* = 12.7, df₁ = 9, df₂ = 1,560, *p* < 0.001). The significant predictors are verbal working memory (*β* = 0.176, *t* = 6.24, *p* < 0.001) and age.

A comparison between the baseline model and Model 2.1 showed a significant improvement in model quality (Δ*R*^2^ = 0.03, *F* = 13.9, *p* < 0.001). A comparison of the baseline model and Model 2.2 did not reveal a significant improvement in model quality (Δ*R*^2^ = 0.003, *F* = 1.81, *p* = 0.144). A comparison between Model 2.1 and Model 3 revealed no further improvement (Δ*R*^2^ = 0.000, *F* = 0.341, *p* = 0.796). Comparing Model 2.2 and Model 3 revealed significant improvement (*ΔR*^2^ = 0.03, *F* = 12.8, *p* < 0.001).

Based on the largest *R*^2^, Model 2.1 was selected as the best-fitting model (see Table 4). Thus, the most significant predictor of narrative skills in different genres is verbal working memory.

**Table 4:** Regression model of scale 4: narrative skills in different genres.

Predictor	Estimate	SE	*t*	*p*	Stand. estimate
Intercept	4.65229	0.79203	5.874	<0.001	
Gender	0.26632	0.15862	1.679	0.093	0.0823
Age	0.03196	0.01087	2.939	0.003	0.0854
Cognitive flexibility	0.01638	0.02915	0.562	0.574	0.0159
Visual working memory	0.00754	0.00424	1.779	0.075	0.0538
Inhibition	−0.02073	0.02635	−0.787	0.432	−0.0203
Verbal working memory	0.12173	0.01933	6.298	<0.001	0.1760

## Discussion

The aim of the present study was to assess the contribution of EF and EC to key aspects of pragmatic competence in children aged 5–8 years. Our data showed that the result depended on EF aspect. Working memory in children from 5 to 8 years old was proved to be the strongest predictor for all the aspects of pragmatic competence. It corresponds with data, reported by V. Bambini and her colleagues in aged people (Bambini et al., 2021). Cognitive flexibility and inhibitory control had significant correlations with pragmatic competence in children, however they did not enhance it. As shown in previously cited studies (Bambini et al., 2021), verbal working memory makes the greatest contribution to pragmatic skills. The impact of EF on such pragmatic ability as narrative construction was also proved in longitudinal study (Oshchepkova and Shatskaya, 2023). The current study demonstrated the same effect on other aspects of pragmatic skills. The comprehension of figurative meaning requires children to retain both instructions and what was said in memory. This is especially important for interpreting the figurative meanings of words and phrases. For the communicative aspect of pragmatics, working memory also proved to be the most significant predictor. In our view, this can be explained by the fact that communication requires holding in memory what exactly the interlocutor has said and constructing one’s response according to his/her remarks. Why both verbal and visual working memory turned out to be the most significant predictors for the use of nonverbal means of communication is less clear to us. It could be explained by the fact, that the production of gestures implies the ability to coordinate their execution with the meaning of the discourse: children with higher WM could be better able to coordinate the two levels. Furthermore, maintaining the motor plan in mind requires WM. We may assume that this is a matter of working memory in general, which allows one to retain the elements of a communicative situation (both verbal and nonverbal) and respond appropriately to this situation. For Scale 4, verbal working memory made the most significant contribution. This confirms previous findings that working memory influences narrative production in preschool children (Veraksa et al., 2019). We can explain this primarily by the fact that verbal working memory makes a substantial contribution both to children’s language development in general and to the development of narrative skills (Gago-Galvagno et al., 2024; Ryabikina and Vasilchenko, 2023). Therefore, verbal working memory remains the strongest predictor of all aspects of pragmatic competence.

As for the impact of EC on pragmatic competence in children with normative development, our data showed that though EC skills correlated significantly with all the aspects of pragmatic competence in children, the impact of EC skills on pragmatics was statistically less significant then the impact of EF. The contribution of EC to pragmatic competence remains debatable, as noted in the literature we reviewed (Beck et al., 2012; Derakhshan et al., 2021). Since significant correlations (*p* < 0.001) were found between all components of EC and pragmatic competence, we may assume that this relationship reflects a specific directionality. It is pragmatic competence—defined as the child’s ability and skill to communicate and engage in discussions with close others—that may shape their ability to recognize and understand emotions (Guseva et al., 2025; Morozova et al., 2025; Pronina et al., 2021; Veraksa et al., 2023). Thus, a correlational study design can only establish a significant relationship between EC and pragmatic competence, whereas the direction of this relationship requires separate investigation and, in this case, cannot be determined.

It was noteworthy that sex differences were revealed for all the aspects of pragmatic skills. It corresponds with the data received for general verbal abilities (Rzhanova et al., 2023) and may respond to educators’ beliefs about communicative strategies of boys vs. girls or reflect social norms and stereotypes about sex differences in communication (Rudnova et al., 2024).

## Conclusion

The children’s pragmatic competence is significantly influenced by EF, particularly verbal and visual working memory. This reflects the importance of these executive aspects for remembering instructions, rules of games, and what a conversation partner said, especially when figurative meanings are involved. To understand indirect meanings accurately, it is essential not only to retain the literal utterance but also to keep the context in memory. The same mechanisms, namely, the integration of verbal content and social context, are also supported, albeit to a lesser degree, by cognitive flexibility and inhibitory control.

One notable limitation is our inability to isolate and assess the specific role of Theory of Mind in shaping pragmatic competence. This remains an important direction for future studies. Furthermore, we could cite the correlational nature of the design, which prevents the assessment of the direction of relations.

## Data Availability

Data supporting the findings of this study are available from the authors upon reasonable request. Requests to access the datasets should be directed to Ekaterina Oshchepkova, maposte06@yandex.ru.
