# Supplementary material for: The contribution of executive functions and emotion comprehension skills to the development of pragmatic competence in 5–8-year-old children
Source: Front Psychol. 2025 Sep 25;16:1659576. doi: 10.3389/fpsyg.2025.1659576 (PMC12507778; doi:10.3389/fpsyg.2025.1659576)
Supplement: Supplementary file 1 [file Table_1.docx]

**Supplementary materials**

**Table 1** Сorrelations for main study variables

|  |  | S1. Discourse comprehension | S2. Communication skills | S3. Nonverbal cues | S4. Discourse styles and genres | Visual working memory | Verbal working memory | Inhibition | Cognitive flexibility | Emotions caused by external factors | Mental state emotions | Reflective-level emotions | Age | Sex |
| --- | --- | --- | --- | --- | --- | --- | --- | --- | --- | --- | --- | --- | --- | --- |
| S1. Discourse comprehension | Pearson's r | — |  |  |  |  |  |  |  |  |  |  |  |  |
|  | df | — |  |  |  |  |  |  |  |  |  |  |  |  |
|  | p-value | — |  |  |  |  |  |  |  |  |  |  |  |  |
|  | Spearman's rho | — |  |  |  |  |  |  |  |  |  |  |  |  |
|  | df | — |  |  |  |  |  |  |  |  |  |  |  |  |
|  | p-value | — |  |  |  |  |  |  |  |  |  |  |  |  |
|  | N | — |  |  |  |  |  |  |  |  |  |  |  |  |
| S2. Communication skills | Pearson's r | 0.766*** | — |  |  |  |  |  |  |  |  |  |  |  |
|  | df | 1840 | — |  |  |  |  |  |  |  |  |  |  |  |
|  | p-value | <.001 | — |  |  |  |  |  |  |  |  |  |  |  |
|  | Spearman's rho | 0.762*** | — |  |  |  |  |  |  |  |  |  |  |  |
|  | df | 1840 | — |  |  |  |  |  |  |  |  |  |  |  |
|  | p-value | <.001 | — |  |  |  |  |  |  |  |  |  |  |  |
|  | N | 1842 | — |  |  |  |  |  |  |  |  |  |  |  |
| S3. Nonverbal cues | Pearson's r | 0.682*** | 0.717*** | — |  |  |  |  |  |  |  |  |  |  |
|  | df | 1840 | 1840 | — |  |  |  |  |  |  |  |  |  |  |
|  | p-value | <.001 | <.001 | — |  |  |  |  |  |  |  |  |  |  |
|  | Spearman's rho | 0.689*** | 0.714*** | — |  |  |  |  |  |  |  |  |  |  |
|  | df | 1840 | 1840 | — |  |  |  |  |  |  |  |  |  |  |
|  | p-value | <.001 | <.001 | — |  |  |  |  |  |  |  |  |  |  |
|  | N | 1842 | 1842 | — |  |  |  |  |  |  |  |  |  |  |
| S4. Discourse styles and genres | Pearson's r | 0.584*** | 0.630*** | 0.601*** | — |  |  |  |  |  |  |  |  |  |
|  | df | 1840 | 1840 | 1840 | — |  |  |  |  |  |  |  |  |  |
|  | p-value | <.001 | <.001 | <.001 | — |  |  |  |  |  |  |  |  |  |
|  | Spearman's rho | 0.573*** | 0.617*** | 0.598*** | — |  |  |  |  |  |  |  |  |  |
|  | df | 1840 | 1840 | 1840 | — |  |  |  |  |  |  |  |  |  |
|  | p-value | <.001 | <.001 | <.001 | — |  |  |  |  |  |  |  |  |  |
|  | N | 1842 | 1842 | 1842 | — |  |  |  |  |  |  |  |  |  |
| Visual working memory | Pearson's r | 0.242*** | 0.198*** | 0.149*** | 0.153*** | — |  |  |  |  |  |  |  |  |
|  | df | 1741 | 1741 | 1741 | 1741 | — |  |  |  |  |  |  |  |  |
|  | p-value | <.001 | <.001 | <.001 | <.001 | — |  |  |  |  |  |  |  |  |
|  | Spearman's rho | 0.224*** | 0.179*** | 0.135*** | 0.143*** | — |  |  |  |  |  |  |  |  |
|  | df | 1741 | 1741 | 1741 | 1741 | — |  |  |  |  |  |  |  |  |
|  | p-value | <.001 | <.001 | <.001 | <.001 | — |  |  |  |  |  |  |  |  |
|  | N | 1743 | 1743 | 1743 | 1743 | — |  |  |  |  |  |  |  |  |
| Verbal working memory | Pearson's r | 0.295*** | 0.253*** | 0.195*** | 0.241*** | 0.386*** | — |  |  |  |  |  |  |  |
|  | df | 1769 | 1769 | 1769 | 1769 | 1730 | — |  |  |  |  |  |  |  |
|  | p-value | <.001 | <.001 | <.001 | <.001 | <.001 | — |  |  |  |  |  |  |  |
|  | Spearman's rho | 0.283*** | 0.241*** | 0.192*** | 0.227*** | 0.402*** | — |  |  |  |  |  |  |  |
|  | df | 1769 | 1769 | 1769 | 1769 | 1730 | — |  |  |  |  |  |  |  |
|  | p-value | <.001 | <.001 | <.001 | <.001 | <.001 | — |  |  |  |  |  |  |  |
|  | N | 1771 | 1771 | 1771 | 1771 | 1732 | — |  |  |  |  |  |  |  |
| Inhibition | Pearson's r | 0.113*** | 0.074** | 0.070** | 0.041 | 0.244*** | 0.171*** | — |  |  |  |  |  |  |
|  | df | 1727 | 1727 | 1727 | 1727 | 1639 | 1668 | — |  |  |  |  |  |  |
|  | p-value | <.001 | 0.002 | 0.003 | 0.088 | <.001 | <.001 | — |  |  |  |  |  |  |
|  | Spearman's rho | 0.128*** | 0.088*** | 0.076** | 0.038 | 0.260*** | 0.190*** | — |  |  |  |  |  |  |
|  | df | 1727 | 1727 | 1727 | 1727 | 1639 | 1668 | — |  |  |  |  |  |  |
|  | p-value | <.001 | <.001 | 0.002 | 0.119 | <.001 | <.001 | — |  |  |  |  |  |  |
|  | N | 1729 | 1729 | 1729 | 1729 | 1641 | 1670 | — |  |  |  |  |  |  |
| Cognitive flexibility | Pearson's r | 0.199*** | 0.134*** | 0.108*** | 0.129*** | 0.424*** | 0.359*** | 0.244*** | — |  |  |  |  |  |
|  | df | 1754 | 1754 | 1754 | 1754 | 1666 | 1692 | 1662 | — |  |  |  |  |  |
|  | p-value | <.001 | <.001 | <.001 | <.001 | <.001 | <.001 | <.001 | — |  |  |  |  |  |
|  | Spearman's rho | 0.191*** | 0.129*** | 0.111*** | 0.133*** | 0.443*** | 0.389*** | 0.261*** | — |  |  |  |  |  |
|  | df | 1754 | 1754 | 1754 | 1754 | 1666 | 1692 | 1662 | — |  |  |  |  |  |
|  | p-value | <.001 | <.001 | <.001 | <.001 | <.001 | <.001 | <.001 | — |  |  |  |  |  |
|  | N | 1756 | 1756 | 1756 | 1756 | 1668 | 1694 | 1664 | — |  |  |  |  |  |
| Emotions caused by external factors | Pearson's r | 0.147*** | 0.108*** | 0.063** | 0.096*** | 0.306*** | 0.250*** | 0.096*** | 0.301*** | — |  |  |  |  |
|  | df | 1711 | 1711 | 1711 | 1711 | 1675 | 1704 | 1617 | 1694 | — |  |  |  |  |
|  | p-value | <.001 | <.001 | 0.009 | <.001 | <.001 | <.001 | <.001 | <.001 | — |  |  |  |  |
|  | Spearman's rho | 0.122*** | 0.086*** | 0.048* | 0.076** | 0.302*** | 0.235*** | 0.087*** | 0.274*** | — |  |  |  |  |
|  | df | 1711 | 1711 | 1711 | 1711 | 1675 | 1704 | 1617 | 1694 | — |  |  |  |  |
|  | p-value | <.001 | <.001 | 0.045 | 0.002 | <.001 | <.001 | <.001 | <.001 | — |  |  |  |  |
|  | N | 1713 | 1713 | 1713 | 1713 | 1677 | 1706 | 1619 | 1696 | — |  |  |  |  |
| Mental state emotions | Pearson's r | 0.120*** | 0.113*** | 0.055* | 0.075** | 0.281*** | 0.158*** | 0.058* | 0.183*** | 0.194*** | — |  |  |  |
|  | df | 1711 | 1711 | 1711 | 1711 | 1675 | 1704 | 1617 | 1694 | 1711 | — |  |  |  |
|  | p-value | <.001 | <.001 | 0.022 | 0.002 | <.001 | <.001 | 0.019 | <.001 | <.001 | — |  |  |  |
|  | Spearman's rho | 0.107*** | 0.097*** | 0.050* | 0.074** | 0.280*** | 0.180*** | 0.059* | 0.181*** | 0.185*** | — |  |  |  |
|  | df | 1711 | 1711 | 1711 | 1711 | 1675 | 1704 | 1617 | 1694 | 1711 | — |  |  |  |
|  | p-value | <.001 | <.001 | 0.039 | 0.002 | <.001 | <.001 | 0.018 | <.001 | <.001 | — |  |  |  |
|  | N | 1713 | 1713 | 1713 | 1713 | 1677 | 1706 | 1619 | 1696 | 1713 | — |  |  |  |
| Reflective-level emotions | Pearson's r | 0.108*** | 0.075** | 0.03 | 0.053* | 0.254*** | 0.221*** | 0.093*** | 0.195*** | 0.168*** | 0.163*** | — |  |  |
|  | df | 1711 | 1711 | 1711 | 1711 | 1675 | 1704 | 1617 | 1694 | 1711 | 1711 | — |  |  |
|  | p-value | <.001 | 0.002 | 0.213 | 0.029 | <.001 | <.001 | <.001 | <.001 | <.001 | <.001 | — |  |  |
|  | Spearman's rho | 0.099*** | 0.069** | 0.033 | 0.053* | 0.246*** | 0.238*** | 0.096*** | 0.209*** | 0.165*** | 0.162*** | — |  |  |
|  | df | 1711 | 1711 | 1711 | 1711 | 1675 | 1704 | 1617 | 1694 | 1711 | 1711 | — |  |  |
|  | p-value | <.001 | 0.004 | 0.176 | 0.03 | <.001 | <.001 | <.001 | <.001 | <.001 | <.001 | — |  |  |
|  | N | 1713 | 1713 | 1713 | 1713 | 1677 | 1706 | 1619 | 1696 | 1713 | 1713 | — |  |  |
| Age | Pearson's r | 0.185*** | 0.138*** | 0.099*** | 0.167*** | 0.482*** | 0.367*** | 0.076** | 0.342*** | 0.283*** | 0.279*** | 0.252*** | — |  |
|  | df | 1840 | 1840 | 1840 | 1840 | 1741 | 1769 | 1727 | 1754 | 1711 | 1711 | 1711 | — |  |
|  | p-value | <.001 | <.001 | <.001 | <.001 | <.001 | <.001 | 0.002 | <.001 | <.001 | <.001 | <.001 | — |  |
|  | Spearman's rho | 0.183*** | 0.142*** | 0.105*** | 0.170*** | 0.491*** | 0.388*** | 0.086*** | 0.349*** | 0.273*** | 0.273*** | 0.252*** | — |  |
|  | df | 1840 | 1840 | 1840 | 1840 | 1741 | 1769 | 1727 | 1754 | 1711 | 1711 | 1711 | — |  |
|  | p-value | <.001 | <.001 | <.001 | <.001 | <.001 | <.001 | <.001 | <.001 | <.001 | <.001 | <.001 | — |  |
|  | N | 1842 | 1842 | 1842 | 1842 | 1743 | 1771 | 1729 | 1756 | 1713 | 1713 | 1713 | — |  |
| Sex | Pearson's r | 0.091*** | 0.085*** | 0.090*** | 0.04 | -0.016 | -0.007 | 0.048* | 0.023 | 0.035 | -0.026 | -0.004 | -0.061** | — |
|  | df | 1840 | 1840 | 1840 | 1840 | 1741 | 1769 | 1727 | 1754 | 1711 | 1711 | 1711 | 1840 | — |
|  | p-value | <.001 | <.001 | <.001 | 0.089 | 0.492 | 0.779 | 0.044 | 0.325 | 0.151 | 0.281 | 0.866 | 0.009 | — |
|  | Spearman's rho | 0.091*** | 0.092*** | 0.090*** | 0.041 | -0.016 | 0.003 | 0.051* | 0.018 | 0.02 | -0.036 | -0.003 | -0.063** | — |
|  | df | 1840 | 1840 | 1840 | 1840 | 1741 | 1769 | 1727 | 1754 | 1711 | 1711 | 1711 | 1840 | — |
|  | p-value | <.001 | <.001 | <.001 | 0.081 | 0.513 | 0.907 | 0.035 | 0.44 | 0.404 | 0.136 | 0.912 | 0.007 | — |
|  | N | 1842 | 1842 | 1842 | 1842 | 1743 | 1771 | 1729 | 1756 | 1713 | 1713 | 1713 | 1842 | — |
| *Note.* * p < .05, ** p < .01, *** p < .001 | | | | | | | | | | | | | | |
